# Supplementary material for: Microsatellite Characterization and Panel Selection for Brown Bear (Ursus arctos) Population Assessment
Source: Genes (Basel). 2022 Nov 19;13(11):2164. doi: 10.3390/genes13112164 (PMC9690282; doi:10.3390/genes13112164)
Supplement: Supplementary file 1 [file genes-13-02164-s001.zip › genes-2028700-supplementary.pdf]

Table S1. Primer sequences, characteristics, and annealing temperatures of the 32 microsatellite loci tested here. Only the selected multiplexes (BEST panels) are indicated for each brown bear (*Ursus arctos*) subspecies.

Locus	Repeat motiv	Forward primer	Reverse primer	Annealing temp (°C)	Dye	Multiplex
G10B [1]	di-	TGCTAATATTTTCTTGAGGACT	AGGACAAATCACAGAAACCT	52.5	HEX	
G10C [1]	di-	GTCTGCAAAAGCAGAAGG (*Uam*)<br>AAAGCAGAAGGCCTTGATTTCCTG (*Uaa*)	AAACACCGAGACAGCAGG (*Uam*)<br>GGGACATAAACACCGAGACAGC (*Uaa*)	52.5	NED	3 - *Uam*
G10H [1]	di-	CAACAAGAAGACCACTGTAA	AGAGACCACCAAGTAGGATA	52.5	6_FAM	
G10L [1]	di-	ACTGATTTTATTCACATTTCCC	GATACAGAAACCTACCCATGCG	57	HEX	
G10M [5]	di-	GTTTGCCTCTTTGKCTACTGGA	CAAATAATTTAAATGCATCCCAGGGG	52.5	6_FAM	
G10P [1]	di-	AGTTTTACATAGGAGGAAGAA	TCATGTGGGGAAATACTCTGAA	52.5	6_FAM	
G10X [1]	di-	CCCTGGTAACCACAAATCTCT	TCAGTTATCTGTGAAATCAAAA	52.5	6_FAM	2 – *Uaa*
G1D [1]	di-	CCATCTCTCTTTTCCTTTAGGG	CTACTCTTCCTACTCTTTAAGAG	52.5	PET	3 - *Uam*
Mu05 [2]	di-	AATCTTTTCACTTATGCCCA	GAAACTTGTTATGGGAACCA	57	PET	
Mu09 [2]	di-	AGCCACTTTGTAAGGAGTAGT	ATATAGCAGCATATTTTTGGCT	52.5	HEX	2 – *Uaa*
Mu10 [2]	di-	ATTCAGATTTCATCAGTTTGACA	TCAGCATAGTTACACAAATCTCC	52.5	NED	
Mu11 [2]	di-	AAGTAATTGGTGAAATGACAG	GAACCCTTCACCGAAAATC	52.5	HEX	3 – *Uam*<br>2 – *Uaa*
Mu15 [2]	di-	CTGAATTATGCAATTAAACAGC (*Uam*)<br>GCCTGACCATCCAACATC (*Uaa*)	AAATAAGGGAGGCTTGGGT	52.5	HEX	
Mu23 [2]	di-	GCCTGTGTGCTATTTTATCC	AATGGGTTTCTTGTTTAATTAC	52.5	6_FAM	2 – *Uaa*
Mu50 [2]	di-	GTCTCTGTCATTTCCCCATC	AACCTGGAACAAAAATTAACAC	57	HEX	3 – *Uaa*
Mu51 [2]	di-	AGCCAGAATCCTAAGAGACCT	AAAGAGAAGGGACAGGAGGTA	52.5	HEX	3 - *Uam*
Mu59 [2]	di-	GCTCCTTTGGGACATTGTAA	TGACTGTCACCAGCAGGAG	57	NED	2 - *Uam*
cxx20 [3]	di-	AGCAACCCCTCCCATTTACT	TTGTCTGAATAGTCCTCTGCG	57	NED	
REN144A06 [3]	di-	TTTTATGGTTGAGTGCTATTCC	GAAATTGGCCACAGTTCCAT	57	6_FAM	
UA03 [4]	tetra-	GCTCCCATAACTGCATAAGGTC	CTGGCTGGCTGGCTAGG	57	6_FAM	1 – *Uaa*
UA06 [4]	tetra-	CCTCACTTAGCAGCCTACTTG	TGCTCTTCTCTTCAAACTGAGC	57	HEX	
UA14 [4]	tetra-	CCACATTACTGCCAGATAGAGC	ACATCAAACACTAATGATGCACTG	57	HEX	1 – *Uaa*
UA16 [4]	tetra-	CCCCCAAGTCAATTTCTAATATG	CCTTTAGTTTAGTGGCCATCAATC	57	HEX	4 – *Uaa*
UA17 [4]	tetra-	AAGGGTCAGAATTAGGTATCTGTC	TGCTATTTCCATCTTCAACCTGAC	57	6_FAM	1 – *Uaa*
UA25 [4]	tetra-	CTCCATTTGGGGTCTGTTGT	GATTGCTTCATGCACGCTTA	57	PET	1 - *Uam*
UA51 [4]	tetra-	ACCACTTTACTTCCTCATGTCTG	GTGAGTTCAAGCACCACGTAG	57	6_FAM	3 – *Uaa*
UA57 [4]	tetra-	ACATCTAGGACCAAGCATTGC	GTCTGCCTCTTAACCATGGC	57	6_FAM	2 - *Uam*
UA63 [4]	tetra-	TATCCACTCACCATCCACCA	CCAGGAAGCGTAACTCCAGA	57	NED	
UA64 [4]	tetra-	CATGCACTCTCTGTATCCTGCT	CCTCTACCCTCTGCCTCGAC	57	PET	2 - *Uam*
UA65 [4]	tetra-	TCAGGGTTCTCCAAAGAAACA	CTGGGCCTCCACTATCATGT	57	6_FAM	4 – *Uaa*
UA67 [4]	tetra-	TCCTGCTTACCGCACTTCTT	GAGGACACCAGCTGTGAGAA	57	NED	2 - *Uam*
UA68 [4]	tetra-	TTCCCAACTTCCAAACACCC	GGTAGGTAAGAAGGCATGCATG	57	HEX	1 – *Uaa*
Amel4/SE47	Sex	AGAGGCAGGTCAGGAAGCAT	CAGCCAAACCTCCCTCTGC	57	6_FAM	1 – *Uam*<br>1 – *Uaa*

[1] loci designed on a genomic library of American black bears (*U. americanus*) [55–57]
[2] loci designed on a genomic library of European brown bears (*U. arctos*) [58, 59]
[3] loci designed on the canid genome [60, 61]
[4] loci developed for High-throughput sequencing (HTS) [36]
[5] loci modified from [59]
